# Identification of Hub Genes Associated With Development and Microenvironment of Hepatocellular Carcinoma by Weighted Gene Co-expression Network Analysis and Differential Gene Expression Analysis

**DOI:** 10.3389/fgene.2020.615308

**Published:** 2020-12-22

**Authors:** Qingquan Bai, Haoling Liu, Hongyu Guo, Han Lin, Xuan Song, Ye Jin, Yao Liu, Hongrui Guo, Shuhang Liang, Ruipeng Song, Jiabei Wang, Zhibo Qu, Huaxin Guo, Hongchi Jiang, Lianxin Liu, Haiyan Yang

**Affiliations:** ^1^Department of Hepatic Surgery, The First Affiliated Hospital of Harbin Medical University, Harbin, China; ^2^Department of Hepatobiliary Surgery, Anhui Province Key Laboratory of Hepatopancreatobiliary Surgery, The First Affiliated Hospital of USTC, Division of Life Sciences and Medicine, University of Science and Technology of China, Hefei, China; ^3^Department of Pediatric Surgery, The Fourth Affiliated Hospital of Jiangsu University, Zhenjiang, China; ^4^Department of Endocrinology, The First Affiliated Hospital of Harbin Medical University, Harbin, China; ^5^Department of Medical Administration, The First Affiliated Hospital of Harbin Medical University, Harbin, China

**Keywords:** hepatocellular carcinoma, differential gene expression analysis, weighted gene co-expression network analysis, tumor microenvironment, overall survival

## Abstract

A further understanding of the molecular mechanism of hepatocellular carcinoma (HCC) is necessary to predict a patient’s prognosis and develop new targeted gene drugs. This study aims to identify essential genes related to HCC. We used the Weighted Gene Co-expression Network Analysis (WGCNA) and differential gene expression analysis to analyze the gene expression profile of GSE45114 in the Gene Expression Omnibus (GEO) database and The Cancer Genome Atlas database (TCGA). A total of 37 overlapping genes were extracted from four groups of results. Kyoto Encyclopedia of Genes and Genomes (KEGG) pathway and Gene Ontology (GO) enrichment analyses were performed on the 37 overlapping genes. Then, we used the STRING database to map the protein interaction (PPI) network of 37 overlapping genes. Ten hub genes were screened according to the Maximal Clique Centrality (MCC) score using the Cytohubba plugin of Cytoscape (including FOS, EGR1, EPHA2, DUSP1, IGFBP3, SOCS2, ID1, DUSP6, MT1G, and MT1H). Most hub genes show a significant association with immune infiltration types and tumor stemness of microenvironment in HCC. According to Univariate Cox regression analysis and Kaplan-Meier survival estimation, SOCS2 was positively correlated with overall survival (OS), and IGFBP3 was negatively correlated with OS. Moreover, the expression of IGFBP3 increased with the increase of the clinical stage, while the expression of SOCS2 decreased with the increase of the clinical stage. In conclusion, our findings suggest that SOCS2 and IGFBP3 may play an essential role in the development of HCC and may serve as a potential biomarker for future diagnosis and treatment.

## Introduction

Hepatocellular carcinoma (HCC) is a common tumor with a high morbidity and mortality rate (Torre et al., 2015). Surgical resection remains the most effective treatment and is widely recommended (Hasegawa et al., 2013). However, the prognosis of HCC is low, as only 10–20% carcinomas can be removed entirely by surgery (Sun et al., 2019). The high recurrence rate remains the most severe challenge for surgical resection, and the 5-year survival rate is only 30–40% (Portolani et al., 2006). Therefore, a thorough study of the dysregulated gene in HCC and building a prognosis model to predict the overall survival (OS) are great significance to improve the prognosis and rehabilitation of patients with HCC.

Weighted gene co-expression network analysis (WGCNA) is an important method to understand gene function and gene association from genome-wide expression. WGCNA can detect co-expression modules of highly related genes and modules of interest related to clinical features (Zhang and Horvath, 2005), which provides a good insight into the function of co-expressed genes and finds genes that play a crucial role in human diseases (Saris et al., 2009; Yang et al., 2014; Li et al., 2018). Also, differential gene expression analysis provides a method for studying the molecular mechanism of genome regulation and detecting quantitative changes in expression levels (Segundo-Val and Sanz-Lozano, 2016). This difference in gene expression can lead to the discovery of potential biomarkers for specific diseases. Therefore, two methods were used to combine the results from WGCNA and differential gene expression analysis to improve the recognition ability of highly related genes as candidate biomarkers.

In our study, the mRNA expression data of HCC in The Cancer Genome Atlas database (TCGA) and Gene Expression Omnibus (GEO) databases were analyzed by WGCNA and differential gene expression analysis. Overlapping genes were obtained from the intersection of the four sets of results. We performed the KEGG pathway and GO enrichment analysis of overlapping genes and constructed a protein-protein interaction (PPI) network to screen hub genes. The hub genes were analyzed by tumor microenvironment analysis, dimension reduction analysis, survival analysis, and clinical information correlation analysis. It provides a potential basis for further understanding of the molecular mechanism, clinical diagnosis, and treatment of HCC.

## Materials and Methods

### Datasets From TCGA and GEO Database

We downloaded the RNA-sequencing dataset of 50 normal liver tissue samples and 374 HCC samples with corresponding clinical data from The TCGA.^1^ Besides, 49 HCC patient samples with prognostic information from the GSE45114 dataset were also downloaded.^2^ As suggested in the edgeR package tutorial (Robinson et al., 2010), genes with low read counts usually do not require further analysis. Therefore, in this study, we maintained genes with CPM (count per million) >1. The Limma package was applied to perform the analysis.^3^ The *p*-value was adjusted by the Benjamini-Hochberg method to control the false discovery rate (FDR). We also used the Limma package to identify the differentially expressed genes given |log2 fold change (FC)| ≥ 1 and false discovery rate (FDR) < 0.05.

### Using WGCNA to Identify Key Co-expression Modules

Co-expression networks facilitate network-based gene screening, which can be used to identify candidate biomarkers and therapeutic targets. We developed the gene expression profile of TCGA-LIHC and GSE45114 into the gene co-expression network using the WGCNA package (8). WGCNA was used to analyze the modules of highly related genes among samples and find the gene modules related to the external traits of samples. In order to construct a scale-free network, pick soft power *β* = 3, and 20 are selected by the picksofhold function. Then, the adjacency matrix is established by the following formula: aij = |Sij|β (aij: adjacency matrix between gene i and gene j, Sij: similarity matrix which is done by Pearson correlation of all gene pairs, β: soft power value), and transformed into a topological overlap matrix (ToM) and a corresponding dissimilarity (1-tom). Then, the hierarchical clustering tree of the 1-tom matrix is constructed to divide the similar gene expression into different gene co-expression modules. To further identify the functional modules in the co-expression network, the module feature association and clinical feature information between the modules were calculated. Therefore, modules with a high correlation coefficient are considered candidate modules related to clinical features and selected for subsequent analysis.

### KEGG Pathway and GO Enrichment Analysis and PPI Network Construction and Hub Genes Selection

We submit differentially expressed RBPs to the STRING database for the KEGG pathway and GO enrichment analysis and protein-protein interaction information (PPI) recognition (Szklarczyk et al., 2019).^4^ The PPI network is further visualized by using the software of Cytoscape 3.7.1. In a co-expression network, Maximal Clique Centrality (MCC) algorithm was used to select the hub genes (Thul and Lindskog, 2018).

### Tumor Microenvironment Analysis

The ESTIMATEscore, immune score, and stromal score were used to analyze the immune cells’ infiltration and stromal cells in different tumors (Yoshihara et al., 2013). The estimated score of the program is used to describe the purity of the tumor. The analysis was based on the expression of data from TCGA (Yoshihara et al., 2013).^5^ Spearman correlation was used to analyze the correlation between hub gene expression and these scores. Six immune subtypes were defined to measure the immune infiltration in the tumor environment (Thorsson et al., 2018). The correlation between the expression of the hub gene and the type of immune infiltrates in the tumor microenvironment was tested by the ANOVA model using the immune subtypes obtained from TCGA-LIHC data. Tumor stemness characteristics extracted from the transcriptome and epigenetics of TCGA tumor samples were used to measure stem-cell-like characteristics of tumor cells (Malta et al., 2018). Spearman correlation analysis was used to detect the correlation between hub gene expression and tumor stemness.

## Results

### Weighted Gene Co-expression Network Analysis

**Figure 1:**
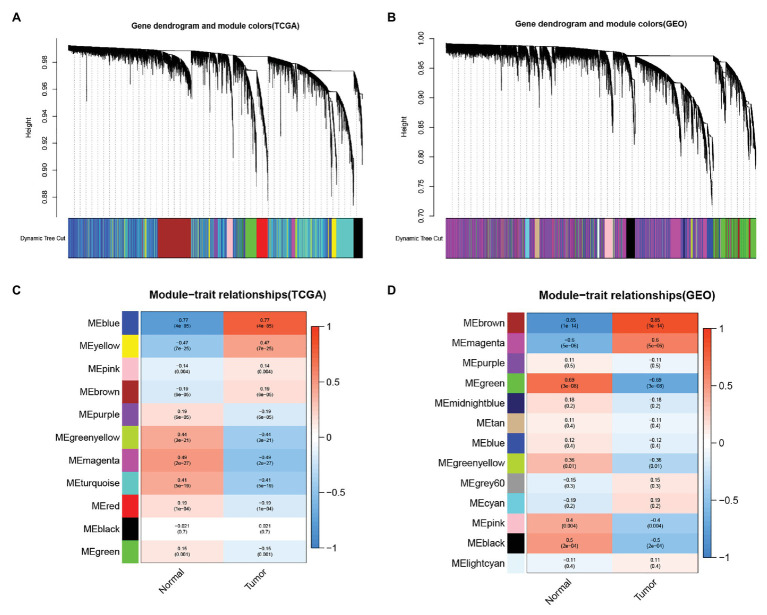
Identify the module information related to clinical. The clustering tree of the co-expression network module is sorted by hierarchical gene clustering based on the 1-tom matrix. Each module is given a different color. **(A)** Gene tree and module color in The Cancer Genome Atlas database (TCGA). **(B)** Gene dendrogram and module color in GSE45114. Module feature relationship. Each row corresponds to a color module, and columns correspond to cancer or normal tissue. Each cell contains the corresponding correlation and value of *p*. **(C)** Module trait relationship in TCGA. **(D)** Module feature relationship inGSE45114.

We analyzed The Cancer Genome Atlas database-Liver hepatocellular carcinoma (TCGA-LIHC) and GSE45114 data sets and constructed a weighted gene co-expression network to obtain functional gene clusters. In this study, 11 TCGA-LIHC modules and 13 GSE45114 modules were found, and each module was assigned colors (Figures 1A,B). We then evaluated the correlation between each module and cancer and normal tissue. The results showed that the blue module in TCGA-LIHC(*r* = −0.77, *p* = 4e-85, Figure 1C) and the brown module in GSE45114(*r* = −0.85, *p* = 1e-14, Figure 1D) had the highest correlation with tumor tissue.

### Acquisition of Differentially Expressed Genes and Overlapping Genes

**Figure 2:**
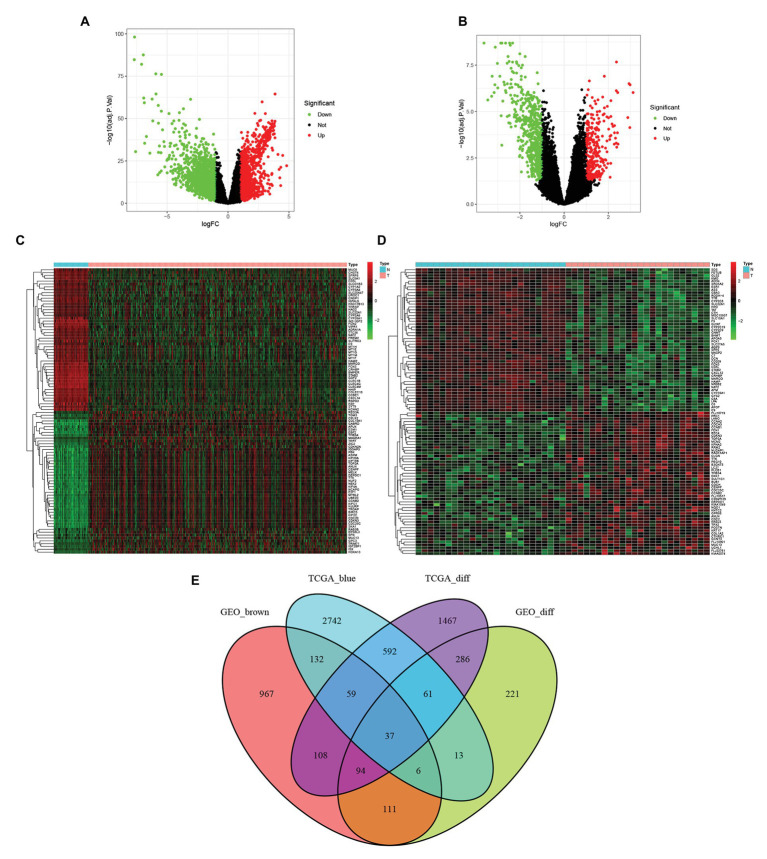
Using | logfc | > 1.0 and adj *p* < 0.05 as cut-off criteria, the differentially expressed genes in TCGA and GSE45114 data sets of Hepatocellular carcinoma (HCC) were identified. **(A)** Volcano maps of differentially expressed genes in the TCGA dataset. **(B)** Volcano map of differentially expressed genes in the GSE45114 dataset. **(C)** Heatmap of 100 differentially expressed genes in the TCGA dataset. **(D)** Heatmap of 100 differential genes in the GSE45114 dataset. **(E)** Venn diagram of gene crossover between differential expression gene list and co-expression module. A total of 37 overlapping genes were located at the intersection of the differential expression gene list and the co-expression module.

Genes differentially expressed in tumor and adjacent normal tissues were screened in the TCGA-LIHC data set (Figure 2A) and GSE45114 data set (Figure 2B). The 100 differentially expressed genes in each data set were mapped into heatmaps to assess the differences in expression between tumor and adjacent normal tissues (Figures 2C,D). The genes in the blue module of TCGA-LIHC data set and the genes ingrown module of GSE45114 and differentially expressed genes in TCGA-LIHC data set and GSE45114 data set were intersected. A total of 37 overlapping genes were extracted for further study (Figure 2E).

### KEGG Pathway and GO Enrichment Analysis of the 37 Overlapping Genes

**Figure 3:**
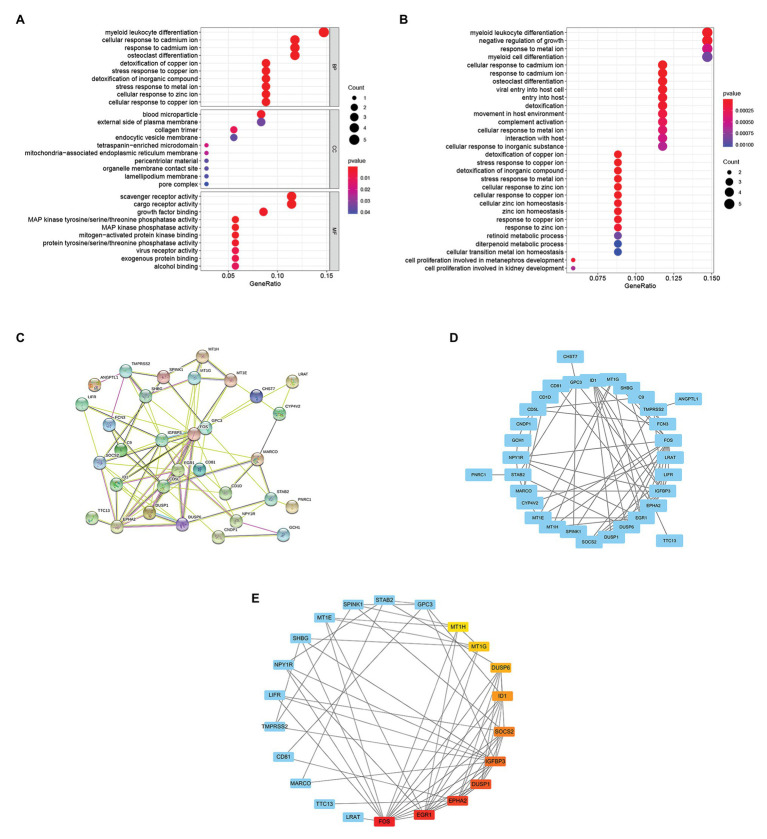
Enrichment analysis of overlapping genes and selection of hub genes. **(A)** Gene Ontology (GO) enrichment analysis for the 37 overlapping genes. **(B)** KEGG pathway enrichment analysis for the 37 overlapping genes. **(C)** The STRING database constructed the PPI network of 37 overlapping genes. **(D)** The PPI network of 37 overlapping genes was compiled by Cytoscape software. The blue nodes represent genes, and the edges represent the interaction between nodes. **(E)** The maximal clique centrality (MCC) algorithm is used to identify hub genes in PPI networks. The edge represents the binding of protein and protein. The red node represents the gene with a high MCC score, while the Yellow node represents the gene with a low MCC score.

To further study the function of 37 overlapping genes, we conducted the Kyoto Encyclopedia of Genes and Genomes (KEGG) pathway and Gene Ontology (GO) enrichment analysis of the 37 overlapping genes. After GO enrichment analysis, the results indicated that 37 overlapping genes in the biological process (BP) were mainly related to myeloid leukocyte differentiation; cellular response to cadmium ion; response to cadmium ion; osteoclast differentiation; cellular response to copper ion; cellular response to zinc ion; stress response to metal ion; detoxification of inorganic compound; stress response to copper ion; and detoxification of copper ion (Figure 3A). The cellular component (CC) of 37 overlapping genes were mainly related in the blood microparticle; external side of plasma membrane; collagen trimer; endocytic vesicle membrane; pore complex; lamellipodium membrane; organelle membrane contact site; pericentriolar material; mitochondria-associated endoplasmic reticulum membrane; and tetraspanin-enriched microdomain (Figure 3A). Through the molecular function (MF) analysis, we found that the 37 overlapping genes were mainly related to scavenger receptor activity; cargo receptor activity; growth factor binding; alcohol binding; exogenous protein binding; virus receptor activity; protein tyrosine/serine/threonine phosphatase activity; mitogen-activated protein kinase binding; MAP kinase phosphatase activity; and MAP kinase tyrosine/serine/threonine phosphatase activity (Figure 3A). Moreover, in the KEGG pathway enrichment analysis, 37 overlapping genes were mainly related to myeloid leukocyte differentiation; negative regulation of growth; response to metal ion; myeloid cell differentiation; cellular response to inorganic substance interaction with host; cellular response to metal ion; complement activation; movement in host environment; detoxification; entry into the host; viral entry into host cell; osteoclast differentiation; response to cadmium ion; and cellular response to cadmium ion (Figure 3B).

### PPI Network Construction and Hub Genes Identification

The PPI network of 37 overlapping genes was established using the STRING database (Figure 3C), and the hub genes were selected from the PPI network by the MCC algorithm of CytoHubba plugin (Figure 3D). The top 10 genes with the highest score of MCC sores were identified as hub genes, including the Fos proto-oncogene (FOS), early growth response 1(EGR1), EPH receptor A2(EPHA2), dual specificity phosphatase 1(DUSP1), insulin like growth factor binding protein 3(IGFBP3), suppressor of cytokine signaling 2(SOCS2), inhibitor of DNA binding 1(ID1), dual specificity phosphatase 6(DUSP6), metallothionein 1G(MT1G), and metallothionein 1H(MT1H; Figure 3E).

### Hub Genes Expressio With Immune Infiltration Types in HCC

**Figure 4:**
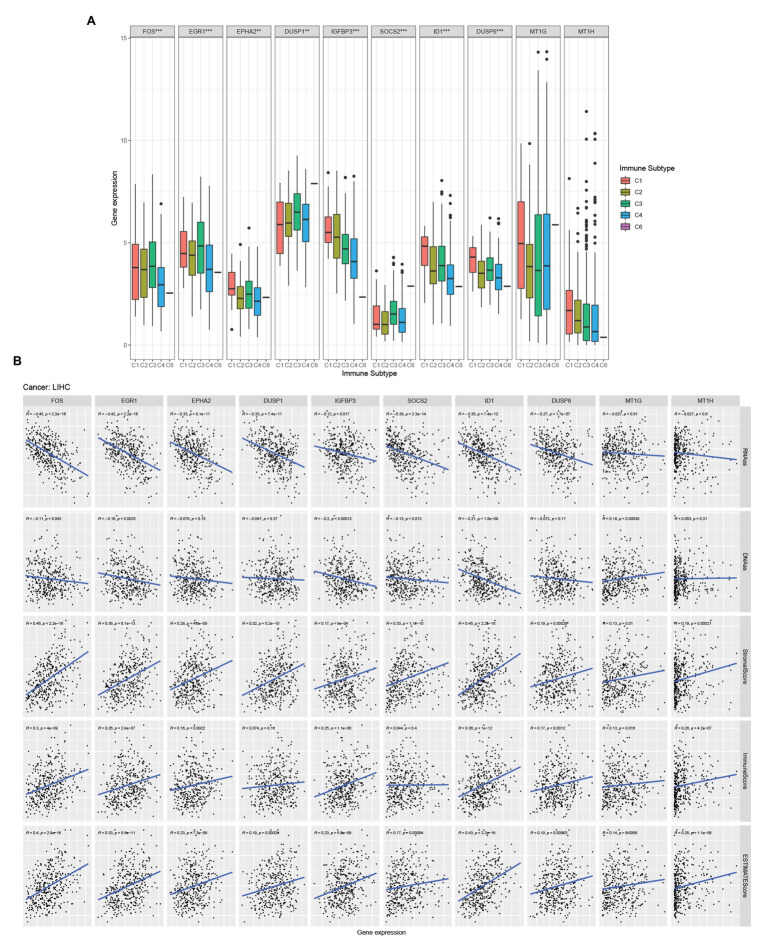
Hub genes and HCC microenvironment analysis. **(A)** Association of hub genes expression with immune infiltrate subtypes in HCC tested with ANOVA. **(B)** Correlation matrixes between hub gene expression and RNAss, DNAss, stromal score, immune score, and Estimate Score. Spearman correlation tests were used for testing.

There are six types of immune infiltration in human tumors: C1 (wound healing), C2 (INF-r dominant), C3 (inflammatory), C4 (lymphocyte depleted), C5 (immunologically quiet), and C6 (TGF*β* dominant; Tamborero et al., 2018). To study the relationship between hub genes and immune components in HCC, we detected the correlation between hub genes expression and immune infiltration types in the TCGA-LIHC data set (Figure 4A). Through the Kruskal test, we found that FOS, EGR1, EPHA2, DUSP1, IGFBP3, SOCS2, ID1, and DUSP6 were related to immune infiltration subtype.

### Hub Genes Expression With HCC Tumor Stemness of the Microenvironment in HCC

Tumor stemness can be measured with RNA stemness score based on the mRNA expression (RNAss) and DNA stemness score (DNAss) based on the DNA methylation pattern (Malta et al., 2018). We examined the relationship between the expression of hub genes and the RNAss, DNAss, StromalScore, ImmuneScore, and ESTIMATEScore of tumor microenvironment in HCC. The RNAss was significantly negatively correlated with FOS, EGR1, EPHA2, DUSP1, IGFBP3, SOCS2, ID1, and DUSP6 (Figure 4B). Besides, FOS, EGR1, IGFBP3, SOCS2, ID1, and MT1G showed a negative correlation with DNAss (Figure 4B). Moreover, FOS, EGR1, EPHA2, IGFBP3, ID1, DUSP6, MT1G, and MT1H were positive with ImmuneScore (Figure 4B). Moreover, all the hub genes were positive with StromalScore and ESTIMATEScore (Figure 4B).

### Expression and Prognostic Values of Hub Genes in HCC

**Figure 5:**
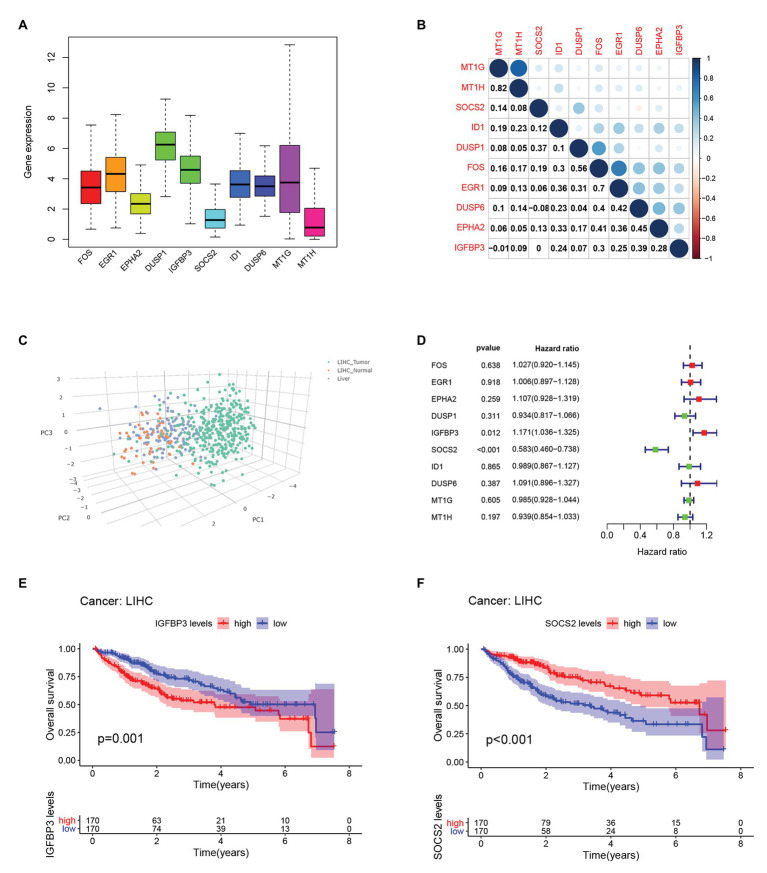
Hub genes expression and survival analysis in HCC.**(A)** Comparison of the expression of each hub gene in HCC.**(B)** Expression correlation of each hub gene in HCC.**(C)** Dimension reduction analysis of all hub genes using The cancer genome atlas database-liver hepatocellular carcinoma (TCGA-LIHC) data set and GTEx data set. **(D)** Univariate Cox regression analysis of OS analysis of hub genes from the TCGA-LIHC database. **(E)** Kaplan-Meier survival estimation of OS analysis of IGFBP3 from the TCGA-LIHC database. **(F)** Kaplan-Meier survival estimation of OS analysis of SOCS2 from the TCGA-LIHC database.

**Figure 6:**
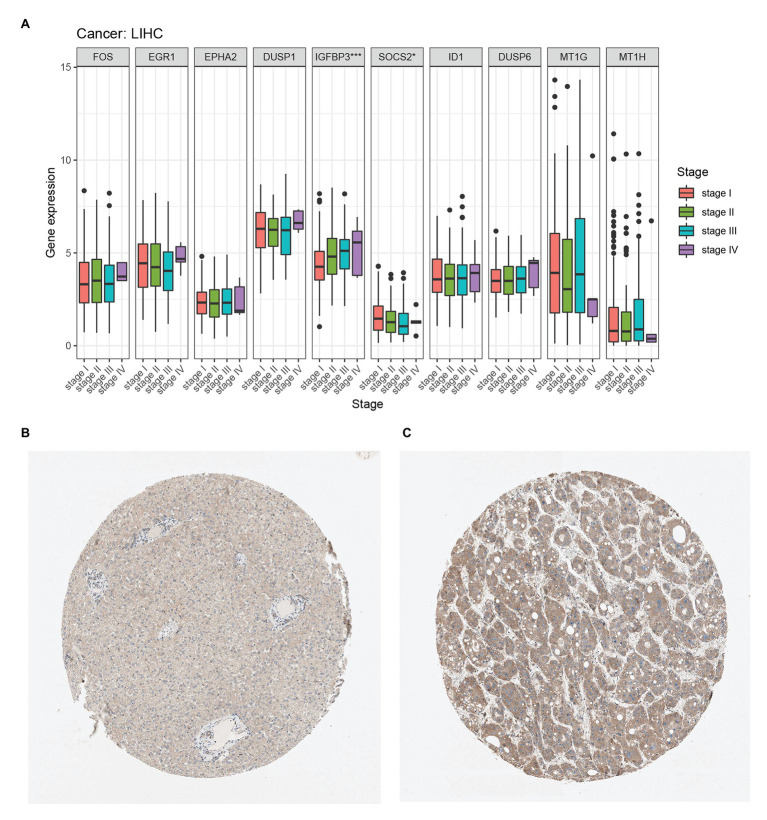
Hub genes clinical stage and immunohistochemistry in HCC. **(A)** Relationship between hub gene expression and clinical stage of HCC from the TCGA-LIHC database. **(B)** Immunohistochemistry of the SOCS2 in HCC tissues from the human protein atlas (HPA) database (staining: Low; intensity: Weak; quantity: >75%). **(C)** Immunohistochemistry of the SOCS2 in normal liver tissues from the HPA database. (staining: Medium; intensity: Moderate; quantity: >75%).

We compared the expression of each hub gene in HCC and found that the expression of DUSP1 was the highest, and the expression of MT1H was the lowest in HCC (Figure 5A). We also compared the expression correlation of each hub gene in HCC, where MT1G and MT1H had the most positive correlation (Figure 5B). The hub genes were also analyzed by dimension reduction using the TCGA-LIHC data set and Genotype-Tissue Expression (GTEx) data set (Figure 5C). All hub genes were evaluated by Univariate Cox regression analysis and Kaplan-Meier survival estimation to study the relationship between gene expression and overall survival. In Univariate Cox regression analysis, IGFBP3 was negatively correlated with OS, while SOCS2 was positively correlated with OS, and other hub genes were not correlated with OS (Figure 5D). Moreover, in Kaplan-Meier survival estimation in patients with survival from 1 to 100 months, patients with high expression of SOCS2 had a better OS, while patients with high expression of IGFBP3 had a poor OS, and other hub genes were not correlated with OS (Figures 5E,F). We also analyzed the relationship between hub gene expression and the clinical stage of HCC. We found that only the expression of IGFBP3 and SOCS2 was related to the clinical stage of HCC, and the expression of IGFBP3 increased with the increase of clinical stage, while the expression of SOCS2 decreased with the increase of the clinical stage (Figure 6A). The protein level of the SOCS2 gene in HCC was significantly higher than that in normal tissues based on the HPA database (Figures 6B,C). However, there is no IGFBP3 protein expression data in the HPA database.

## Discussion

Hepatocellular carcinoma is one of the most common malignant tumors. It has a high incidence rate, high mortality rate, and poor prognosis. Only a few tumors can be completely removed by surgery. However, the treatment of HCC has improved. The 5-year survival rate is only 30–40%. Therefore, more precise molecular targets are needed to determine the prognosis and progress of HCC.In our study, 37 differentially expressed genes in the same most significant gene functional cluster were identified using WGCNA and gene expression analysis in the TCGA-LIHC data set and GSE45114 data set. Then, we performed the KEGG pathway and GO enrichment analysis of the 37 genes and found that they play an important role in many biological processes. Besides, the top 10 HCC related hub genes were screened according to MCC scores from the CytoHubba plugin in Cytoscape, including FOS, EGR1, EPHA2, DUSP1, IGFBP3, SOCS2, ID1, DUSP6, MT1G, and MT1H. We analyzed the relationship between hub genes expression and immune response and tumor microenvironment in HCC. We found that the expression of FOS, EGR1, EPHA2, DUSP1, IGFBP3, SOCS2, ID1, and DUSP6 would affect the immune infiltration types. The expression level of FOS, EGR1, DUSP1, and SOCS2 in C3(inflammatory) immune infiltration subtype was higher than that in other immune infiltration subtypes, while the expression level of EPH2, IGFBP3, ID1, and DUSP6 in the C1 (wound healing) immune infiltration subtype was high. A significant positive correlation was found between all hub genes and Stromal Score and ESTIMATE Score in HCC. Besides, FOS, EGR1, EPHA2, IGFBP3, ID1, DUSP6, MT1G, and MT1H showed a significantly positive correlation with the Immune Score. However, FOS, EGR1, IGFBP3, SOCS2, ID1, and MT1G in HCC were negatively related to DNAss. We also found that FOS, EGR1, EPHA2, DUSP1, IGFBP3, SOCS2, ID1, and DUSP6 were negatively related to RNAss. We analyzed the expression correlation of hub genes and found that most of the hub genes were positive correlation expressions. In HCC tissues, the expression of MT1H was the lowest, and DUSP1 was the highest, and all their expression was upregulated in HCC compared with adjacent liver tissue. We evaluated all hub genes by Univariate Cox regression analysis and Kaplan-Meier survival estimation. IGFBP3 was correlated with the poor OS, while SOCS2 was correlated with the good OS, and other hub genes were not correlated with OS. Finally, immunohistochemistry was verified that the expression of SOCS2 in HCC was higher than in the liver.

Insulin-like growth factor-binding protein 3 is also known as IGFBP-3. IGFBP-3 is one of six IGF binding proteins (IGFBP-1 to IGFBP-6) that have highly conserved structures. The imbalance of IGFBP-3 is associated with many cancers (Baxter, 2014). However, the regulatory mechanism of IGFBP-3 on tumors remains unclear. In breast cancer (Yu et al., 1998; Sheen-Chen et al., 2009), pancreatic cancer, (Xue et al., 2008), and clear cell renal cell carcinoma (Takahashi et al., 2005), the high expression of IGFBP-3 is associated with poor prognosis. However, IGFBP-3 promoter methylation and decreased expression lead to poor prognosis of non-small cell lung cancer (Chang et al., 2002). Besides, circulating levels of IGFBP-3 are associated with increased risk of some cancers, but the results vary from site to site (Renehan et al., 2004). IGFBP-3 protein level decreased in the process of prostate cancer metastasis from benign to malignant (Miyake et al., 2000). The risk of colon cancer was positively correlated with plasma IGFBP-3 (Palmqvist et al., 2002). In our study, This is consistent with our analysis that IGFBP-3 is highly expressed in HCC, and high IGFBP-3 expression is associated with poor prognosis of HCC.IGFBP-3 was highly expressed in HCC, and the high expression of IGFBP-3 is associated with poor prognosis of HCC.

Suppressor of cytokine signaling 2, also known as SOCS2, is a member of the STAT-induced STAT inhibitor (SSI), also known as suppressor of cytokine signaling (SOCS), family. In cancer, low SOCS2 gene expression was associated with breast cancer, lung cancer, hepatocellular carcinoma, and ovarian cancer (Wikman et al., 2002; Sutherland et al., 2004; Farabegoli et al., 2005; Haffner et al., 2007; Qiu et al., 2013; Zhu et al., 2013). In contrast, SOCS2 was highly expressed in bone marrow cells of patients with chronic myeloid leukemia (CML; Schultheis et al., 2002). The expression of SOCS2 in breast cancer decreased with the increase of tumor grade (Sasi et al., 2010). High SOCS2 expression was an independent predictor of good prognosis in breast cancer (Haffner et al., 2007; Sasi et al., 2010). In HCC, the downregulation of SOCS2 was significantly associated with the late stage of TNM (Qiu et al., 2013). The low expression of SOCS2 in primary prostate tissue was associated with an increased incidence of postoperative metastasis and decreased SOCS2 levels during prostate cancer (Hendriksen et al., 2006; Iglesias-Gato et al., 2014). Overexpression of SOCS2 inhibited the proliferation of Caco-2 colon cancer cell line (Miller et al., 2004). This evidence supports that SOCS2 generally limits tumor growth, which is consistent with our conclusion.

Our study suffers from some limitations. Although we have used a variety of bioinformatics methods to analyze the diagnostic genes of HCC, the molecular mechanism of genes affecting the prognosis and survival of HCC patients needs to be further studied through a series of experiments.

## Conclusion

Our study combined WGCNA with differential gene expression analysis, further verified by immune infiltration-type analysis and tumor stemness of microenvironment analysis, and we obtained the survival-related genes IGFBP3 and SOCS2, which may predict the prognosis of HCC.

## Data Availability Statement

Publicly available datasets were analyzed in this study. This data can be found here: TCGA and GSE45114. The data that support the findings of this study are available from the corresponding authors upon reasonable request.

## Author Contributions

QB, HY, and LL designed the research. QB carried out the research and analyzed the data. HL, HlL, HyG, XS, YL, YJ, HrG, SL, RS, JW, ZQ, HxG, and HJ analyzed the data and wrote the paper. All authors contributed to the article and approved the submitted version.

### Conflict of Interest

The authors declare that the research was conducted in the absence of any commercial or financial relationships that could be construed as a potential conflict of interest.

## Footnotes

^1^https://portal.gdc.cancer.gov/

^2^https://www.ncbi.nlm.nih.gov/geo/query/acc.cgi?acc=GSE45114

^3^http://www.bioconductor.org/packages/release/bioc/html/limma.html

^4^http://www.string-db.org/

^5^https://bioinformatics.mdanderson.org/estimate/

## Abbreviations

HCC: Hepatocellular carcinoma
WGCNA: Weighted gene co-expression network analysis
GEO: Gene expression omnibus
TCGA: The cancer genome atlas database
MCC: Maximal clique centrality
OS: Overall survival
PPI: Protein interaction
HPA: Human protein atlas
TCGA-LIHC: The cancer genome atlas database-liver hepatocellular carcinoma
KEGG: Kyoto encyclopedia of genes and genomes
BP: Biological process
CC: Cellular component
MF: Molecular function
GTEx: Genotype-tissue expression
FDR: False discovery rate
FC: Fold change
CPM: Count per million
ToM: Topological overlap matrix.
